# Simultaneous Separation and Determination of Nine Active Ingredients in Sanyetangzhiqing by Cyclodextrin-Modified Micellar Electrokinetic Capillary Electrophoresis-Diode Array Detector

**DOI:** 10.1155/2023/4840457

**Published:** 2023-07-12

**Authors:** Shanshan Wang, Rui Zhou, Kunze Du, Ye Shang, Jun He, Jin Li, Yaqi Yao, Yan-xu Chang

**Affiliations:** ^1^State Key Laboratory of Component-Based Chinese Medicine, Tianjin University of Traditional Chinese Medicine, Tianjin 301617, China; ^2^Tianjin Key Laboratory of Phytochemistry and Pharmaceutical Analysis, Tianjin University of Traditional Chinese Medicine, Tianjin 301617, China; ^3^School of Chinese Materia Medica, Tianjin University of Traditional Chinese Medicine, Tianjin 301617, China

## Abstract

A simple and sensitive strategy using cyclodextrin-modified micellar electrokinetic chromatography with diode array detector was developed and applied for the simultaneous separation and determination of nine components in Sanyetangzhiqing (SYTZQ), a hypoglycemic and hypolipidemic agent. Several important parameters affecting separation performance were evaluated and optimized using single variable methods. Under the optimal conditions, baseline separation of the nine components, including four flavonoids (hyperoside, isoquercitrin, quercetin-3-*O*-glucuronoside, and astragalin), four phenolic acids (chlorogenic acid, rosmarinic acid, salvianolic acid B, and lithospermic acid), and a monoterpenoids (paeoniflorin), were achieved in less than 16 min. The correlation coefficients of the calibration curves were over 0.9996 for all the analytes. Intraday and interday precisions ranged from 0.4% to 4.8% and 1.7% to 5.0%, respectively. Recoveries of analytes varied from 95.3% to 105%. Validation results as well as the application to analyse SYTZQ samples demonstrated the applicability of the proposed method and thus provided an effective tool for the quality control of SYTZQ. Moreover, with the advantages of short time consuming, low energy consumption, high efficiency, and low cost, this method has laid a foundation for the determination and quality evaluation of multicomponents in Chinese herbal compounds.

## 1. Introduction

In recent years, the incidence of diabetes, a metabolic disease whose main feature is the increase of blood glucose level, is increasing year by year [1]. Owing the advantages of precise curative effect, safety, and stability, traditional Chinese medicines (TCMs) preparation has aroused widespread attention [2–4]. In order to lower the blood glucose level of patients, Academician Boli Zhang proposed a Chinese herbal compound preparation called Sanyetangzhiqing (SYTZQ), which consisted of *Folium Mori* (*Morus alba* L.), *Crataegi Folium* (*Crataegus pinnatifida Bge.var. major* N.E.Br. *or Grataegus pinnatifida Bge.*), *Lotus Leaf* (*Nelumbo nucifera* Gaertn.), *Salvia Miltiorrhizae Radix Et Rhizoma* (*Salvia miltiorrhiza* Bge.), and *Paeoniae Radix Rubra* (*Paeonia ladiflora* Pall. or *Paeonia veitchii* Lynch) [5]. Relevant pharmacological experiments indicated a wide range of pharmacological effects of this compound preparation, including antihyperlipidemia, antihyperglycemia, and antioxidative stress [6–8]. As a new patent drug for diabetes, SYTZQ has entered phase II clinical trials; preclinical studies showed significant reduction of abnormal glucose and lipid levels in KK-AY model mice with genetic type II diabetes mellitus [9–11]. However, due to the differences of four characters, five tastes, and channel distributions, TCM always plays monarch, minister, assistant, and guide roles in preparation compatibility, the mechanism is not clear, and pharmacodynamic substances are complex. Therefore, it is urgent to clarify the mechanism and pharmacodynamic material basis of SYTZQ and establish a perfect quality evaluation and analysis method.

Content determination of SYTZQ components is an important basis for its quality evaluation. So far, qualitative and quantitative analysis methods for various components in SYTZQ, such as salvianolic acid B, chlorogenic acid, and paeoniflorin, have been established, mainly including liquid chromatography (LC) and LC tandem mass spectrometry (MS), and a quantitative fingerprint based on the quality-derived design concept has been constructed [12–14]. However, there are few reports related to the simultaneous determination of multicomponent in SYTZQ. Given that, it is necessary to establish an accurate and efficient technique of multicomponent determination to improve the quality analysis.

Capillary electrophoresis (CE) is a miniaturized separation technique which presents several advantages: reduced use of reagents, especially organic solvents, which decreased environmental pollution and cost, minimal consumption of samples, high separation efficiency, and high versatility with respect to the composition of the background electrolyte (BGE) and thus could adapt to the different nature of compounds [15]. As a common detection method, CE was often used in the study of TCM and relevant compound preparations. Among the operational modes, micellar electrokinetic chromatography (MEKC) was one of the most popular and flexible modes that combining the principles and advantages of chromatography and CE, by which means it further extends the range of electrophoretic separation to neutral compounds [16–19].

However, in MEKC, hydrophobic or cationic solutes tend to be totally incorporated into the micelle or to strongly interact with the micelle and therefore cannot be separated with simple micellar solution [20]. The cyclodextrin-modified MEKC method uses cyclodextrin (CD) together with an ionic micelle solution and has resolved this problem by establishing two pseudostationary phases in the electrolyte. The use of CD in MEKC could improve the separation of highly hydrophobic solutes, and careful modulation of system selectivity may provide a good resolution of even very demanding analytes [21], which show great advantages in analyzing the complex system of TCM. A cyclodextrin-modified mixed micellar electrokinetic capillary chromatography method was developed simultaneously separating and determinating three huperzine alkaloids, namely, Huperzine A, Huperzine B, and Huperzine C in Huperzia serrata [22]. In this way, tedious sample preparation was avoided and satisfactory resolutions were obtained. By using this method, five anthraquinones with similar structures were also well separated in Rhubarb in a short time [23].

Herein, we focus our attention on the development of a simple and sensitive CD-MEKC method for determining the content of potential antidiabetic components in SYTZQ to provide basis for its quality evaluation research. Considering the complexity of Chinese medicine components, MEKC mode has been employed and sodium dodecyl sulfate (SDS) together with hydroxypropyl-*β*-cyclodextrin (HP-*β*-CD) was used as the pseudostationary phase. The effects of some typical parameters such as pH and concentration of the running buffer, concentration of three additives (SDS, HP-*β*-CD, and ACN), and separation voltage were examined and optimized. Finally, the developed MEKC-DAD method was validated and applied to the determination of nine components in SYTZQ, making it a multicomponent quality evaluation system for TCM and its compound preparations. To our knowledge, this is the first reported study using cyclodextrin-modified MEKC-DAD for the analysis of SYTZQ and also a method that is capable for simultaneously quantitatively analyzing the most kinds of components.

## 2. Experiments

### 2.1. Chemicals and Reagents

The standard compounds, paeoniflorin, chlorogenic acid, rosmarinic acid, hyperoside, isoquercitrin, quercetin-3-*O*-glucuronoside, salvianolic acid B, lithospermic acid, and astragalin (purity > 98%), were purchased from Chengdu Desite Biotechnology Co., Ltd. (Chengdu, China). Analytical grade sodium dodecyl sulfate (SDS) and hydroxypropyl-*β*-cyclodextrinthe (HP-*β*-CD) were procured from Solarbio Biotechnology Co., Ltd. (Beijing, China). Sodium dihydrogen phosphate (NaH_2_PO_4_), disodium hydrogen phosphate (Na_2_HPO_4_), sodium hydroxide (NaOH), and orthophosphoric acid (H_3_PO_4_) (85%) were supplied by Kemiou Chemical Reagent Co., Ltd. (Tianjin, China). HPLC-grade methanol and acetonitrile (ACN) were obtained from Thermo Fisher Scientific (MA, USA). Ultrapure water was purified by the Milli-Q academic water purification system (Millipore, MA, USA).

Five batches of SYTZQ (tablets) all came from the Institute of Chinese Medicine, Tianjin University of Traditional Chinese Medicine, and reviewed by Researcher Yanxu Chang.

### 2.2. Apparatus

All the CE experiments were performed on an Agilent 7100 CE system equipped with a diode array detector (Waldbronn, Germany). Agilent ChemStation software was used to control the instrument and analyze the resulting data. The pH of electrophoretic buffers was adjusted by a Mettler-Lido FE20 pH meter (Shanghai, China). Samples were vortexed using a XW-80A apparatus (Shanghai, China). An ultrasonic cleaning machine (KQ-250E, Kunshan, China) and two balances (BP121S, Sartorius, Germany, and AX205, Mettler Toledo, Switzerland) were also used.

### 2.3. CE Procedures

Sample analysis was performed in an uncoated fused-silica capillary column (Ruifeng, Hebei, China) with a total length of 60 cm (effective length 52 cm) × 50 *μ*m I.D. New capillary was preconditioned for the first time by sequentially flushing with 1 M NaOH (30 min), 0.1 M NaOH (30 min), and deionized water (30 min) at 935 mbar. Every day, before starting the experiments, the capillary was rinsed successively at 930 mbar with 1 M NaOH, 0.1 M NaOH, and deionized water for 10 min, respectively, and then conditioned with background electrolyte (BGE) for 10 min.

Subsequently, the sample was hydrodynamically injected at 50 mbar for 5 s. The separation was carried out with BGE (30 mM phosphate buffer containing 40 mM SDS, 7.5 mM HP-*β*-CD, and 10% ACN, pH 7.0). The electrophoretic separation was set at −20 kV, and the temperature was controlled at 22°C. Between runs, the column was flushed with 0.1 M NaOH, deionized water, and BGE for 3 min, respectively. And at the end of each day, the capillary was washed successively with 0.1 M NaOH and deionized water for 10 min at 930 mbar.

### 2.4. Preparation of Standard and Quality Control Samples

Paeoniflorin and salvianolic acid B were, respectively, weighed at 5.0 mg and individually dissolved with methanol at a final concentration of 5.0 mg/mL as stock solution. Standard stock solutions of chlorogenic acid, rosmarinic acid, hyperoside, isoquercitrin, quercetin-3-*O-*glucuronoside, lithospermic acid, and astragalin were prepared with methanol to a final concentration of 2.0 mg/mL. Ultimately, a series of concentrations of the linear standard solutions with 50% methanol were obtained.

The SYTZQ samples with removing coatings were grinded into powder and passed through an 80-mesh sieve. Then, the powder was accurately weighed 200 mg to 10 mL flask and diluted with 50% methanol to volume. The mixtures performed the ultrasonic extraction of 45 min at 360 W. After cooling, the solution was required to be compensated with 50% methanol for weightlessness. Subsequently, the extracting solution was centrifuged at 14000 r/min for 10 min and then stored at 4°C for later use.

## 3. Results and Discussion

### 3.1. Optimization of BGE Composition

Using micellar as pseudostationary phases, analytes are separated by differential partitioning between the micelle phase and the aqueous phase in MEKC [21]. On account of the characteristics of surfactants, both ionic compounds and neutral compounds could be separated by MEKC, which is especially suitable for the analysis of complex compounds extracted from TCMs [24]. Thus, in this paper, the MEKC mode of CE was selected for detection and separation of the components in SYTZQ. To obtain the optimal analysis results, several factors that greatly influenced the separation were studied.

#### 3.1.1. The Selection of pH

**Figure 1:**
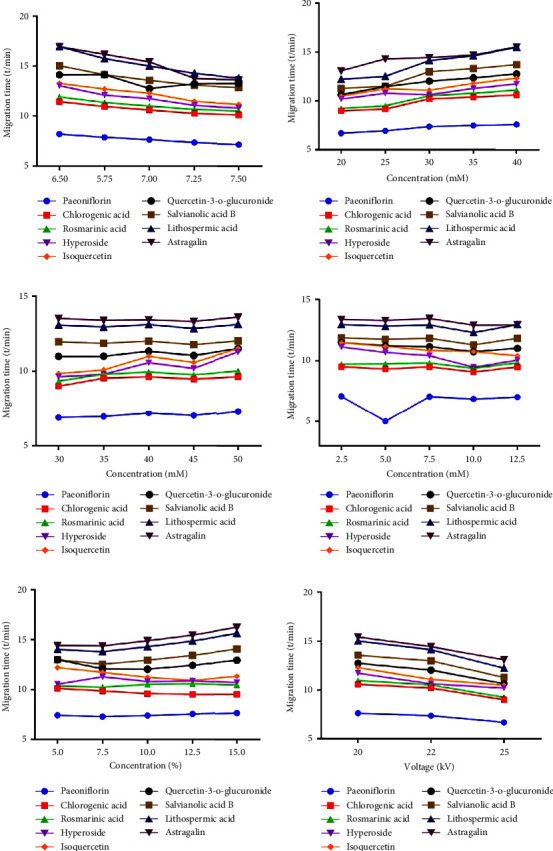
Effects of each parameter on migration time of nine compounds: (a) buffer pH, (b) phosphate concentration, (c) SDS concentration, (d) HP-*β*-CD concentration, (e) ACN content, and (f) separation voltage.

PH, a key factor affecting the electrophoretic mobility of compounds, was firstly investigated by varying from 6.5 to 7.5 with phosphate buffer solutions at 30 mM containing 40 mM SDS, 7.5 mM HP-*β*-CD, and 10% ACN. As seen in Figure 1(a), expected decrease in migration time with increasing pH was observed in the studied range, owing to the increased electroosmotic flow (EOF). Unfortunately, the shortening of migration time affected the compounds' resolution. Results showed that the resolution between peaks was optimum at pH 7.0. When pH was lower than 7.0, the resolution between rosmarinic acid and hyperoside (pH 6.5) or quercetin-3-*O*-glucuronoside and salvianolic acid B (pH 6.75) was poor. However, when pH exceeded higher than 7.0, the augmented EOF caused migration time to decrease, as well as the resolution. Collectively, phosphate buffer at pH 7.0 was further employed to yield better results.

#### 3.1.2. The Selection of Buffer Concentration

The effects of various buffer concentrations (20–40 mM) were then investigated. Generally, a higher buffer concentration provides a better buffering capacity, which in turn affects the separation behavior including resolution, migration time, and peak shape, and also could improve the assay reproducibility [25]. As shown in Figure 1(b), the migration time was raised with increasing buffer concentration. This might be due to the inverse proportion between EOF and ionic strength [26], which extends the migration time and generally improves the separation of analytes with similar electrophoretic migration rates [27]. When the concentration reached up to 30 mM, the resolution and detection sensitivity declined, and peak deformation occurred because of stronger Joule heating. Finally, 30 mM was selected due to symmetrical peak shape and high resolution.

#### 3.1.3. The Selection of SDS Concentration

In MEKC, SDS was often added into running buffer to provide a micellar pseudostationary phase, and the distribution of analytes between SDS and aqueous phase may have a visible effect on resolution and migration time. So, SDS concentration was vital for the separation of analytes. In this paper, SDS concentration was set as 30, 35, 40, 45, and 50 mM. It was found that the resolution increased as SDS concentration increased from 30 to 40 mM and then dropped at 50 mM (Figure 1(c)). Baseline separation could not be obtained between chlorogenic acid, rosmarinic acid, hyperoside, and isoquercitrin when SDS concentration was 30 or 35 mM. When the concentration of SDS was 40 mM, baseline separation of all the nine analytes was achieved and good peak shapes were obtained. However, the resolution decreased when SDS concentration was further increased to beyond 40 mM. This might be because the high concentration of SDS could result in strong interaction between micelle and analytes [28], which reduced the separation efficiency. In consideration of the resolution and peak shape, 40 mM was selected as the optimal concentration for SDS.

#### 3.1.4. The Selection of HP-*β*-CD Concentration

CD could selectively form inclusion complexes with various molecules due to its unique cavity structure, which increases the mobility difference of analytes and improves the resolution during electrophoresis [29, 30]. Actually, the intermolecular interaction between CD and analytes is also a sweeping effect, and the secondary stacking can be realized by micelle in the form of pseudostationary phase. Therefore, the synergistic action of CD and micelle would play a significant influence on resolution and enrichment. In addition, it has been reported that HP-*β*-CD could increase the inclusion of original cavity structure because of the hydroxypropyl group [31]. In view of the above, the effect of HP-*β*-CD was examined by changing the concentration from 2.5 to 12.5 mM with 40 mM SDS and 10% ACN in 30 mM phosphate buffer. According to the results, when CD concentration was 2.5 mM, the peaks of most compounds were overlapped. When the concentration increased to 7.5 mM, resolutions were satisfactory and the peak shapes of all analytes were acceptable. While at the concentration of 10 mM, resolutions decreased again. This might be due to the increased concentration of cyclodextrin which led to the change of interaction with SDS micelles. Taking peak shapes, migration time, and resolutions into consideration, 7.5 mM HP-*β*-CD was selected for subsequent experiments.

#### 3.1.5. The Selection of ACN Content

Liposoluble components could not effectively dissolve in simple buffer solution; to improve the solubility of insoluble substances and ensure the homogeneity of the solution, organic modifiers were frequently used [32]. The function of different concentrations of ACN (5%, 7.5%, 10%, 12.5%, and 15%) on resolution was investigated. In accordance with the results depicted in Figure 1(e), migration times and resolutions of analytes increased with increasing the ACN contents. When the content was 5% and 7.5%, poor resolution accompanied with substance interfering was observed. As the content of ACN was increased to 15%, baseline separation of all analytes was obtained. This might be related to the decrease of EOF and change of SDS micelles. Higher ACN content led to poorer peak shapes and longer migration times for the nine components, and baseline also became worse when the ACN content exceeded 10%. Ultimately, 10% was selected as the optimized ACN content.

#### 3.1.6. The Selection of Separation Voltage

Separation voltage has a direct influence on migration time, resolution, system current, and peak shapes, so voltage ranging from 20 to 25 kV was tested in the last stage. The low voltage (20 kV) resulted in an extended migration time, while resolution was not significantly improved. And, nonsurprisingly, with the high separation voltage (25 kV), though migration time was decreased, Joule heating and current value were increased. Thus, 22 kV was selected in view of separation efficiency, migration time, and current value.

Consequently, according to the abovementioned, the optimized conditions were as follows: 40 mM SDS and 7.5 mM HP-*β*-CD were contained in BGE (30 mM phosphate buffer, pH 7.0), 10% ACN was added as organic modifier, and the separation voltage was fixed at 22 kV.

### 3.2. Method Validation

The performance of the developed MEKC-DAD method for determination of nine active components in SYTZQ was estimated under optimum conditions. The correlation coefficients of calibration curves were all greater than 0.9996. LOD determined at a signal-to-noise ratio (SNR) of 3 was 1.2–3.5 *μ*g/mL, and LOQ determined at a SNR of 10 was 3.7–11.7 *μ*g/mL (LOQ. At three different spiked concentration levels, the RSDs on intraday precision and interday precision ranged from 0.4 to 4.8% and 1.7% to 5.0%, respectively. Recovery experiment was also carried out and the results were 95.3–104%. The results of 24-hour stability showed that the remaining values of all compounds were 96.1–105%, and the RSD values were 1.0–4.9%. All the data (listed in Tables 1 and 2) indicated that the proposed method provided good performances in terms of linearity, LOD, LOQ, precision, stability, and recovery for the simultaneous analysis of the nine components in SYTZQ.

For comparison, several qualitative and quantitative methods for analyzing SYTZQ reported in recent years were summarized, mainly involving HPLC and UPLC methods (Table 3). Thereinto, compared with HPLC, UPLC required less time to realize simultaneous quantification of several compounds in SYTZQ [12–14]. However, large consumption of organic solvent and high operating cost were significant challenges for LC techniques. As we all know, CE is an efficient, green, and stable analysis technique and MEKC mode can be adapted to the sample analysis of a variety of substrates, and it only needs trace organic solvents to meet the rapid determination of multiple components. The method developed herein could realize simultaneous determination of nine compounds with good resolution (*R* ≥ 1.5) in 15 minutes, which even exceeds the separation efficiency of the UPLC method used in [12] (the optimum on resolution among all published methods). Simultaneously, of nine compounds that have been quantified accurately, isoquercitrin, quercetin-3-*O*-glucuronoside, lithospermic acid, and astragalin were quantified for the first time. Besides, there was no requirement for complex sample pretreatment and mobile phase dominated by organic solvent, which was conforming to the concept of green and ecological development. Therefore, compared with other methods, the method developed in this research showed obvious advantages in the quantifying number of components, analysis time, and consumption of organic solvents.

### 3.3. Application to the Determination of the Nine Components in SYTZQ

**Figure 2:**
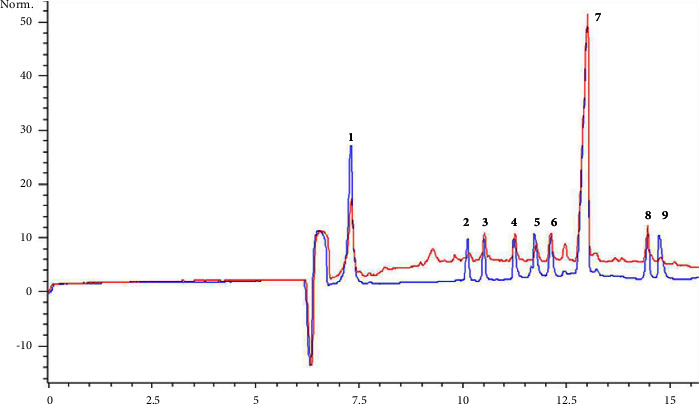
Capillary electropherogram of nine mixed standards (blue) and SYTZQ (red): (1) paeoniflorin, (2) chlorogenic acid, (3) rosmarinic acid, (4) hyperoside, (5) isoquercitrin, (6) quercetin-3-*O*-glucuronoside, (7) salvianolic acid *B*, (8) lithospermic acid, and (9) astragalin.

**Figure 3:**
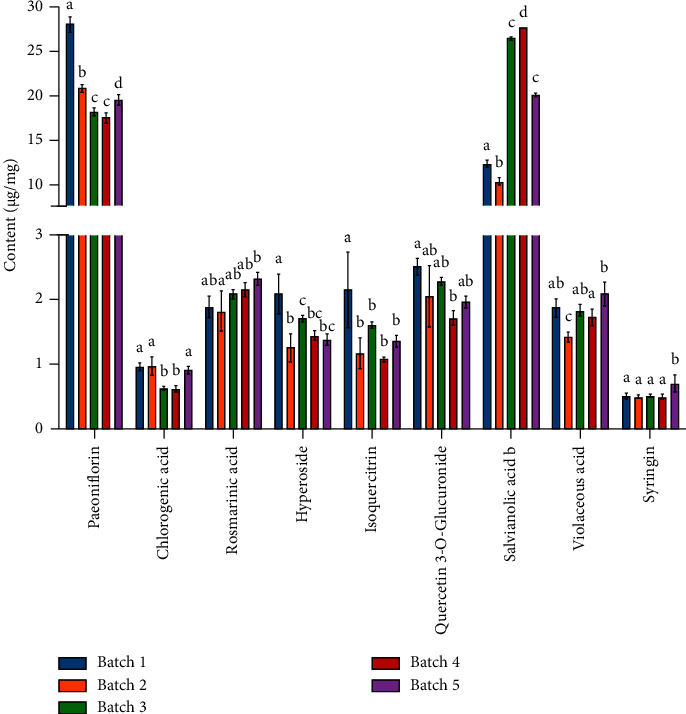
Contents of nine compounds in five batches of SYTZQ. Note: different letters indicate significant differences (*P* < 0.05).

The established method was successfully applied for the determination of nine components (paeoniflorin, salvianolic acid B, chlorogenic acid, rosmarinic acid, hyperoside, isoquercitrin, quercetin-3-*O*-glucuronoside, lithospermic acid, and astragalin) in SYTZQ. By comparing the migration time and spiking standards with sample solutions, the target peaks in sample solutions were identified. Electropherograms of the sample and mixed reference solution are shown in Figure 2, and the quantitative results are shown in Figure 3. It was worth noting that the content of target components varies in different batches of SYTZQ, especially paeoniflorin and salvianolic acid B, and significant differences were indicated according to the statistical analysis. This might be because the quality of original medicinal materials was different and the production time span was large.

## 4. Conclusion

A simple, rapid, and sensitive cyclodextrin-modified micellar electrokinetic capillary electrophoresis method for separation and determination of nine components in SYTZQ was established, and the multicomponent quality evaluation system was formed in this research. The method was optimized and validated, and shorter separation time (16 min), higher sensitivity, and reproducibility were obtained under the optimized conditions. Meanwhile, the analysis of isoquercitrin, quercetin-3-*O*-glucuronoside, lithospermic acid, and astragalin in SYTZQ using CE has been quantified for the first time. Compared with the common quality analysis methods of TCM as well as its compound preparations, the current MEKC-DAD method greatly reduced the organic solvents' consumption and improved the resolution of analytes, which is considered to be a safe, rapid, accurate, and green analysis technology.

## Figures and Tables

**Table 1:** Calibration curves, linearity range, LOD, LOQ, and recoveries of nine compounds.

Compounds	Calibration curve	*R* ^2^	Linearity range (*μ*g/mL)	LOQ (*μ*g/mL)	LOD (*μ*g/mL)	Recovery (%)	RSD (%)
Paeoniflorin	*y* = 303*x* − 3.67	0.9998	50–1000	11.7	3.5	104	2.1
Chlorogenic acid	*y* = 0.57*x* − 0.40	0.9999	5–100	4.6	1.4	99.3	4.4
Rosmarinic acid	*y* = 0.55*x* − 0.19	0.9999	5–100	3.7	1.1	100	2.3
Hyperoside	*y* = 0.71*x* + 0.18	0.9999	5–100	4.2	1.2	95.3	2.2
Isoquercitrin	*y* = 0.76*x* + 0.16	0.9997	5–100	4.7	1.4	105	1.8
Quercetin-3-*O*-glucuronoside	*y* = 0.69*x* − 0.47	0.9999	5–100	4.4	1.3	98.2	2.7
Salvianolic acid B	*y* = 673*x* − 10.64	0.9997	50–1000	5.8	1.7	101	2.7
Lithospermic acid	*y* = 0.74*x* − 0.19	0.9997	5–100	4.0	1.2	103	4.4
Astragalin	*y* = 0.88*x* − 0.54	0.9996	5–100	4.9	1.5	103	1.5

*n = 6.*

**Table 2:** Intraday precision, interday precision, accuracy, and stability of nine compounds.

Compounds	Concentration (*μ*g/mL)	Intraday		Interday		Stability	
		Accuracy (%)	RSD (%)	Accuracy (%)	RSD (%)	Remains (%)	RSD (%)
Paeoniflorin	10	98.3	0.4	97.9	1.7	104	4.0
	20	101	1.5	101	2.6	100	2.0
	50	102	4.5	105	4.1	98.1	2.7

Chlorogenic acid	100	103	4.3	104	4.5	102	4.7
	200	104	4.3	103	3.4	102	2.9
	500	98.5	4.8	100	4.3	104	4.2

Rosmarinic acid	20	99.6	4.0	102	4.4	105	4.6
	40	105	2.6	105	2.9	104	1.0
	100	101	3.1	104	4.8	97.4	3.6

Hyperoside	10	105	2.1	103	3.3	102	3.6
	20	100	4.8	105	4.8	102	4.5
	50	101	4.0	100	4.1	104	2.7

Isoquercitrin	10	99.8	1.5	99.8	4.6	104	3.4
	20	103	2.0	101	5.0	104	3.4
	50	102	4.6	105	4.2	99.0	3.3

Quercetin-3-*O*-glucuronoside	10	98.8	2.0	98.4	4.2	105	2.9
	20	96.1	2.3	99.1	4.7	96.1	4.9
	50	102	4.3	105	4.4	98.3	3.1

Salvianolic acid B	10	98.9	4.6	98.8	4.2	97.0	4.8
	20	104	3.4	102	3.0	102	1.1
	50	96.0	3.4	100	4.4	98.6	4.5

Lithospermic acid	10	100	3.7	97.7	3.9	106	3.9
	20	104	4.5	102	4.2	101	4.9
	50	105	4.6	104	4.9	98.8	3.0

Astragalin	10	104	3.8	104	3.4	101	3.5
	20	99.9	1.6	99.0	1.9	99.6	2.3
	50	103	4.0	102	2.8	103	4.3

*n = 6.*

**Table 3:** Comparison of the analysis method with reported assays.

Components	Number	Method	Organic solvent (mL)	Analysis time (min)	Reference
Chlorogenic acid, paeoniflorin, rutin, hyperoside, quercetin-3-*O*-glucuronoside, and salvianolic acid B	6	HPLC	22.4	56	[13]
Nuciferine, paeoniflorin, salvianolic acid B, hyperoside, and rutin	5	HPLC	26	26	[14]
Paeoniflorin, nuciferine, rutin, and hyperoside	4	UPLC	4.8	16	[12]
Paeoniflorin, chlorogenic acid, rosmarinic acid, hyperoside, isoquercitrin, quercetin-3-*O*-glucuronoside, salvianolic acid B, lithospermic acid, and astragalin	9	MEKC-DAD	0.1	15	This work

## Data Availability

All data generated during the study are available from the corresponding author upon request.
